# A Role of IL-17 in Rheumatoid Arthritis Patients Complicated With Atherosclerosis

**DOI:** 10.3389/fphar.2022.828933

**Published:** 2022-02-08

**Authors:** Jiexin Wang, Linxi He, Weihong Li, Shangbin Lv

**Affiliations:** Basic Medical College, Chengdu University of Traditional Chinese Medicine, Chengdu, China

**Keywords:** rheumatoid arthritis, atherosclerosis, vascular smooth muscle cells, vascular endothelial cells, interleukin-17, inflammation

## Abstract

Rheumatoid arthritis (RA) is mainly caused by joint inflammation. RA significantly increases the probability of cardiovascular disease. Although the progress of RA has been well controlled recently, the mortality of patients with RA complicated with cardiovascular disease is 1.5–3 times higher than that of patients with RA alone. The number of people with atherosclerosis in patients with RA is much higher than that in the general population, and atherosclerotic lesions develop more rapidly in patients with RA, which has become one of the primary factors resulting in the death of patients with RA. The rapid development of atherosclerosis in RA is induced by inflammation-related factors. Recent studies have reported that the expression of IL-17 is significantly upregulated in patients with RA and atherosclerosis. Simultaneously, there is evidence that IL-17 can regulate the proliferation, migration, and apoptosis of vascular endothelial cells and vascular smooth muscle cells through various ways and promote the secretion of several cytokines leading to the occurrence and development of atherosclerosis. Presently, there is no clear prevention or treatment plan for atherosclerosis in patients with RA. Therefore, this paper explores the mechanism of IL-17 in RA complicated with atherosclerosis and shows the reasons for the high incidence of atherosclerosis in patients with RA. It is hoped that the occurrence and development of atherosclerosis in patients with RA can be diagnosed or prevented in time in the early stage of lesions, and the prevention and treatment of cardiovascular complications in patients with RA can be enhanced to reduce mortality.

## Introduction

Rheumatoid arthritis (RA) is a progressive systemic inflammatory disease with unknown etiology, which affects about 0.2–1% of adults worldwide for a long time (Gabriel, 2001; Helmick et al., 2008; Myasoedova et al., 2010; Roth et al., 2017). RA is characterized by inflammatory changes in the joints, resulting in local swelling, pain, and stiffness, usually accompanied by the formation of autoantibodies, such as rheumatoid factor or antinitrate antibodies. Long-term inflammatory stimulation often causes damage and deformity of diseased joints and finally loss of labor ability (Scott et al., 2010; Lillegraven et al., 2012), which greatly impacts social and economic development. What is more severe is that RA, as an autoimmune inflammatory disease, causes local lesions in joint and leads to cardiovascular diseases, including atherosclerosis, cerebrovascular diseases, heart failure, and peripheral vascular diseases (Wolfe et al., 1994; del Rincón et al., 2001). Cardiovascular disease is one of the major reasons for the increase in mortality of patients with RA. Some studies (Crowson et al., 2013; Winchester et al., 2016) have indicated that the mortality of patients with RA with cardiovascular disease is 1.5–3 times higher than that of patients with RA alone. Hence, the study on the mechanism of cardiovascular disease in RA is particularly essential to reduce the mortality of patients with RA. In previous studies, many factors causing cardiovascular disease in RA were found, such as hypertension (McEntegart et al., 2001; Erb et al., 2004), abnormal lipid metabolism (Situnayake and Kitas, 1997), obesity (Stavropoulos-Kalinoglou et al., 2007), smoking (Gerli et al., 2005), and inflammation, among which inflammatory changes are the core link of cardiovascular disease in RA and play an essential role in the occurrence, development, and outcome of the disease. Inflammation is a defense response of the body to stimulation; it is a highly complex process involving many cytokines that cause pathological changes in the lesion site and even the whole body, such as RA. Some studies have discovered several cytokines and chemokines in the synovial tissue of patients with RA. Under the action of these cytokines, dendritic cells (DCs) were activated (Khan et al., 2009); activated DCs expressed several interleukin (IL)-23 (Duvallet et al., 2011) and further stimulated T-cells to differentiate into helper T-cells 17 (TH17), and activated TH17 began expressing IL-17. IL-17 is a cytokine that acts on blood vessels and cardiomyocytes, aggravating inflammation, blood clotting, and thrombosis (Robert and Miossec, 2017). Therefore, blocking the expression of IL-17 or preventing its binding to the receptor may be key to treating cardiovascular diseases in patients with RA.

IL-17 refers to a single cytokine IL-17A or IL-17 family of cytokines composed of IL-17A, IL-17B, IL-17C, IL-17D, IL-17E (IL-25), and IL-17F. There is a large amount of IL-17 expression in the synovial tissue of RA (Chabaud et al., 1999), indicating that IL-17 plays a potential role in the pathogenesis of RA. This view has been verified by different experimental models of arthritis and supported by several human *in vitro* experiments (van den Berg and Miossec, 2009). Subsequently, due to the common pathogenesis of cardiovascular dysfunction and immune diseases (Abou-Raya and Abou-Raya, 2006; Prasad et al., 2015), IL-17 has the same effects on atherosclerosis and chronic inflammation (Ross, 1999; Hansson and Libby, 2006); thus, IL-17 may be involved in both processes. As an increasing number of chronic inflammatory diseases target IL-17 (Beringer et al., 2016), it is crucial to explore the positive or negative effects and related results of IL-17 in RA and cardiovascular diseases.

In view of the high mortality rate of patients with RA with cardiovascular disease, the occurrence and development of coronary heart disease caused by atherosclerosis is the major reason for its high mortality (Kitas et al., 1998; Van Doornum et al., 2002; Kitas and Erb, 2003). Therefore, it is imperative to reveal its pathogenesis to block the occurrence of lesions and decrease mortality. By reviewing previous studies on RA and atherosclerosis, this study shows the core mechanism of the high incidence of cardiovascular disease in patients with RA and provides a theoretical basis for blocking the occurrence and development of cardiovascular disease in patients with RA.

## Inflammation is Key to Atherosclerosis in Patients With RA

In previous studies, many factors were found to cause RA atherosclerosis, such as hypertension, abnormal lipid metabolism, obesity, smoking, and inflammation. Some studies have indicated that (Anyfanti et al., 2020) the increased incidence of hypertension in patients with RA is accompanied by vascular endothelial cell dysfunction, which is usually regarded as a precursor for hypertension. Vascular endothelial cell dysfunction eventually increases the prevalence of atherosclerosis. Regarding abnormal lipid metabolism, previous studies have indicated that elevated levels of low-density lipoprotein can cause cardiovascular disease, and high-density lipoprotein has a protective effect on atherosclerosis. Recent studies have shown that (García-Gómez et al., 2014) the effects of low-density lipoprotein and high-density lipoprotein vary from the actual data related to blood lipids in patients with RA. However, they also prove that abnormal lipid metabolism does increase the risk of atherosclerosis in patients with RA. There is a high amount of adipose tissue in patients with obesity. These adipose tissues act as energy storage organs and can be regarded as a complex dynamic endocrine organ that can secrete a large number of adipose factors (Kershaw and Flier, 2004). Some of these lipokines (such as chemotactic protein, lipoprotein troponin 2, vaspin, and omentin-1) exhibit strong immunomodulatory activity in the pathogenesis of RA (Xie and Chen, 2019). Simultaneously, RA correlates highly with the occurrence and development of atherosclerosis (Tsuji et al., 2001; Hemdahl et al., 2006; Folkesson et al., 2007; Yamawaki et al., 2010; Duan et al., 2011; Kadoglou et al., 2011; Yamawaki et al., 2011; Sivalingam et al., 2017; Wu et al., 2020), but its molecular and physiological mechanism is still unclear. Smoking is seen as a risk factor for RA development, and it is also a significant risk factor for cardiovascular disease in patients with RA (Salonen and Salonen, 1991; Diez-Roux et al., 1995; Silman et al., 1996; Wolfe, 2000; McEntegart et al., 2001; Padyukov et al., 2004; Goodson et al., 2005; Maradit-Kremers et al., 2005). Smoking can promote the manufacture of antinucleotide peptide autoantibodies in susceptible individuals with RA carrying HLA-DRB1 alleles, which cause the aggravation of inflammation and autoimmune diseases (Klareskog et al., 2006; Linn-Rasker et al., 2006), resulting in atherosclerosis (Kraan et al., 1998; Rantapää-Dahlqvist et al., 2003; Vossenaar and van Venrooij, 2004). From the above discussion, it is evident that traditional factors, whether hypertension, obesity, abnormal lipid metabolism, or smoking, will cause abnormal autoimmunity, causing the aggravation of vascular lesions in patients with RA.

Additionally, inflammation is considered the key mechanism of atherosclerosis. Previous studies have suggested that atherosclerosis is caused by the accumulation of lipids in the arterial wall, but new studies suggest that atherosclerosis is inextricably related to inflammation (Libby et al., 2009). Inflammation and autoantibodies may be involved in the occurrence and development of atherosclerotic diseases (Frostegård, 2013; Iseme et al., 2017). Alternatively, inflammation also plays an essential role in inducing plaque erosion and plaque stability, so the development of anti-inflammatory drugs to stabilize plaque is an option for preventing coronary artery disease (CAD) (Everett et al., 2013). The physiology and pathology of atherosclerosis are related to inflammatory lesions of RA synovium in many aspects. Macrophages, helper T-cells (TH1), tumor necrosis factor α (TNF- α), IL-6, and matrix metalloproteinases (MMPs) are all involved in the process (Pasceri and Yeh, 1999). Past studies have indicated that the immune system plays a double-edged role in atherosclerosis development. TH1CD4+ lymphocytes accelerate the formation of atherosclerosis, whereas regulatory T-cells can inhibit atherosclerosis by secreting cytokines, such as transforming growth factor (TGF)-α and IL-10. Changes in the types of cytokines cause an imbalance in T-cell subsets, thereby affecting the disease progression (Ait-Oufella et al., 2006). Additionally, infiltration, accumulation, and oxidation of low-density lipoprotein in the intima of blood vessels can cause inflammation of the arterial wall (Hansson, 2005), Oxidation of low-density lipoprotein results in the expression of adhesion molecules and inflammation-related factors in endothelial cells (Dai et al., 2004); leucocytes infiltrate into the intima through adhesion molecules and differentiate into macrophages under the action of cytokines, such as macrophage colony-stimulating factor and growth factors (Smith et al., 1995; Hansson, 2005). Macrophages, endothelial cells, apoptotic foam cells, and lipid fragments form plaques, leading to narrowing and closure of arteries (Hansson, 2005). Under the action of enzymes, such as inflammatory factors and MMPs, plaques may become unstable or even ruptured (Finn et al., 2010), and plaque rupture may cause thrombosis and blood flow blockage. Blockages of the heart and cerebral arteries cause heart attacks and strokes, respectively (Jones et al., 2003; Hansson, 2005).

In the early stage of RA disease, due to the reduction in the immune system’s self-tolerance, various autoantibodies activate the immune system, resulting in immune cells infiltrating into the synovium of the joint. This process involves the participation of many cytokines, including TNF-α and IL. The levels of TNF-α, IL-17, IL-6, and IL-1 β in serum of patients with RA and cardiovascular disease were increased in varying degrees. These cytokines also participate in the activation of endothelial cells and vascular smooth muscle cells (VSMC), which is the key process in the formation of pannus in synovial tissue during RA lesions and also contributes to the pathogenesis of atherosclerotic heart disease.

## Bone Destruction and Synovial Hyperplasia in RA Are Mediated by IL-17-Guided RA (Figure 1)

**FIGURE 1:**
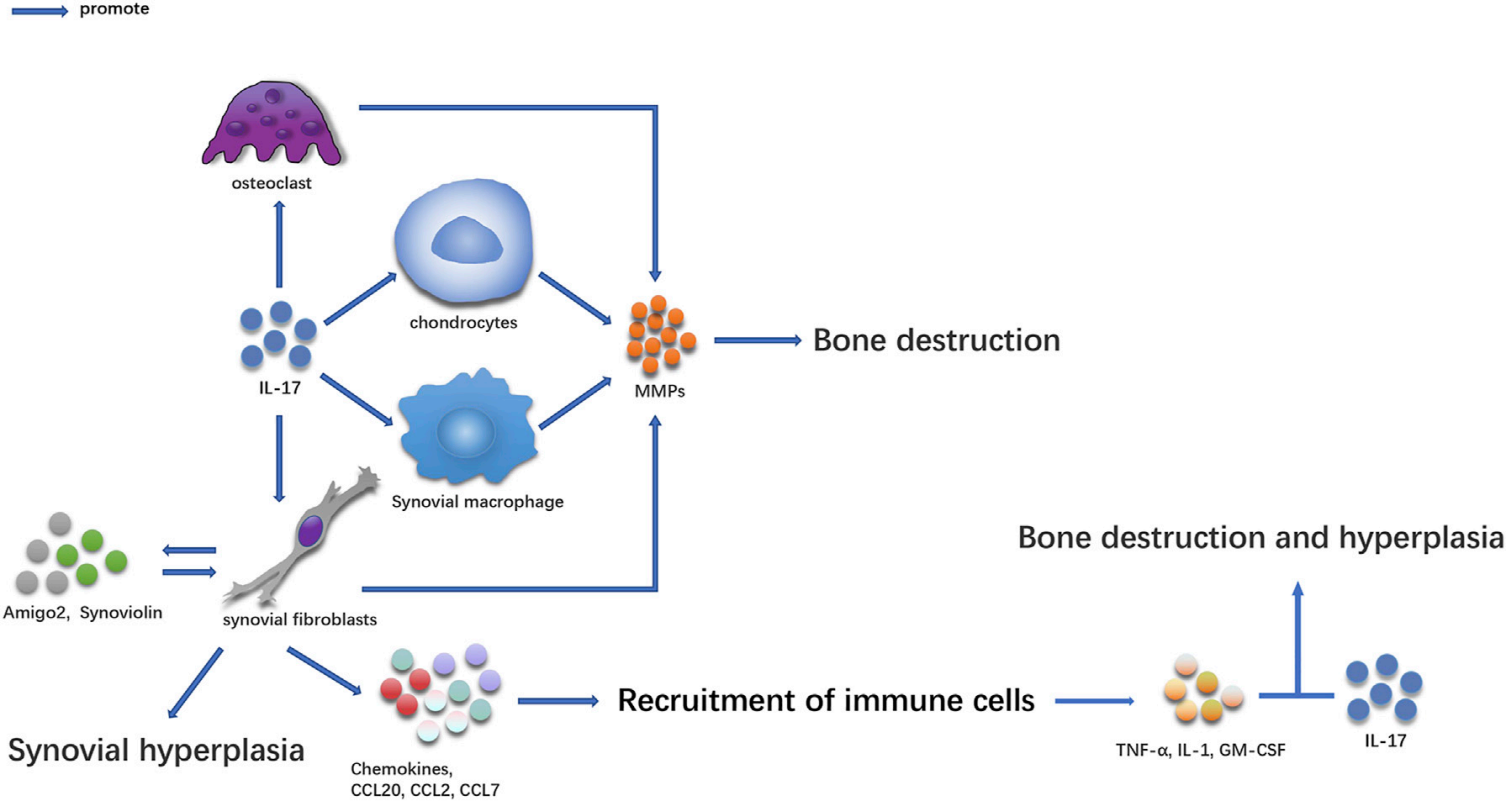
The effect of IL-17 on bone in RA patients.

IL-17-secreting cells were first discovered in the synovium of patients with RA. Some studies have shown that there are many IL-17 and Th17 cells in serum and synovial supernatant of inflammatory joints in patients with RA (Kirkham et al., 2006; Shen et al., 2009; Gullick et al., 2010; Leipe et al., 2010; Raychaudhuri et al., 2012; Gullick et al., 2013). A large amount of IL-17 was also manufactured in peripheral blood mononuclear cells (PBMC) (Kim et al., 2005), and its concentration was higher than that in healthy people (Chabaud et al., 1999; Ziolkowska et al., 2000; Li et al., 2013). Several studies have proven that the content of IL-17 in synovial tissue and serum is directly related to the degree of RA joint injury (Kirkham et al., 2006; Schofield et al., 2016; Siloşi et al., 2016; Lee and Bae, 2017; Costa et al., 2019). The increase in IL-17 levels in serum, synovial fluid, and PBMC is related to the expression of C-reactive protein, erythrocyte sedimentation rate, and rheumatoid factor (Roşu et al., 2012; El-Maghraby et al., 2019). IL-17 participates in the occurrence and development of the disease and plays an essential role in the pathogenesis of the disease (Figure 1) (Raza et al., 2005; Kokkonen et al., 2010).

### IL-17 Causes Bone Destruction at the Lesion Site

In the pathogenesis of RA, IL-17 can cause an imbalance between osteoblasts and osteoclasts in several ways, which often occur in the pathological process of RA (Gravallese and Schett, 2018). The activity of osteoclasts in subchondral, trabecular, and cortical bone eroded areas increased in the joint where IL-17 was highly expressed (Lubberts et al., 2002; Lubberts et al., 2003; Lubberts et al., 2004). IL-17 causes bone destruction by increasing osteoclast formation induced by NF-KB ligand-receptor activator (RANKL) (Lubberts et al., 2004; Adamopoulos et al., 2010). IL-17 can also promote the release of MMPs in synoviocytes and chondrocytes, causing joint injury (Koenders et al., 2005; Agarwal et al., 2008). Collagen-induced arthritis (CIA) is a commonly used animal model for studying RA (Wu et al., 2018), High levels of IL-17 were found in CD4+T-cells and GDT cells in the joints of CIA mice (Pöllinger et al., 2011). Th17 cells transferred to subarticular cartilage osteoclasts to express IL-17, showing that IL-17 was involved in the bone destruction of CIA. Additionally, local injection of IL-17 into the knee joint aggravated arthritis and joint injury during CIA development (Lubberts et al., 2003). Local injection of adenovirus vector expressing mouse IL-17 into the joint can also accelerate the development of CIA and inflammation (Lubberts et al., 2002). Treatment using soluble IL-17 receptor fusion protein or anti-IL-17 antibody can enhance the severity of arthritis, cartilage injury, and bone loss (Lubberts et al., 2001; Bush et al., 2002; Lubberts et al., 2004; Pöllinger et al., 2011). IL-17 can also promote the production of collagen-specific T-cells and collagen-specific IgG2a and participate in CIA occurrence and development (Nakae et al., 2003). Anti-IL-17 can decrease the production and recruitment of inflammatory cells in CIA (Chao et al., 2011; Li et al., 2014).

### IL-17 can Cause Synovium and Bone Hyperplasia in Diseased Joints

IL-17 can also cause synovial and bone hyperplasia while aggravating the inflammatory response of diseased joints. IL-17 promotes the proliferation and survival of synovial cells and induces synovial hyperplasia by inducing the expression of anti-apoptotic molecules, such as synovial protein and amigo2 (Toh et al., 2010; Lee et al., 2013; Benedetti et al., 2016). In the absence of osteoclasts, IL-17, and TNF promote bone proliferation by inducing osteogenic differentiation of mesenchymal stem cells (Osta et al., 2014). IL-17 induces the recruitment of T-cells and other immune cells by inducing the expression of neutrophil chemokine, CC chemokine ligand 20 (CCL20), CCL2, and CCL7 (Hattori et al., 2015). When several immune cells are recruited into the synovium, inflammation in this site is aggravated, and a specific pro-inflammatory cytokine environment is formed, which in turn promotes inflammatory synergism between IL-17 and other cytokines (such as TNF, GM-CSF, or IL-1) and aggravates bone destruction and proliferation in the diseased site. The combination of anti-IL-1 and anti-IL-17 antibodies can effectively inhibit bone and cartilage injury, downregulate the expression of IL-1b, IL-6, IL-17, interferon-gamma (IFN-γ), RANKL, and MMP-3 (Zhang et al., 2013; Li et al., 2014), and decrease the inflammatory changes of the lesion site. Therefore, IL-17 can participate in chronic lesions of RA in several ways.

## IL-17 Is Involved in the Occurrence and Development of Atherosclerosis by Acting on Vascular Endothelial Cells and VSMC (Figure 2)

**FIGURE 2:**
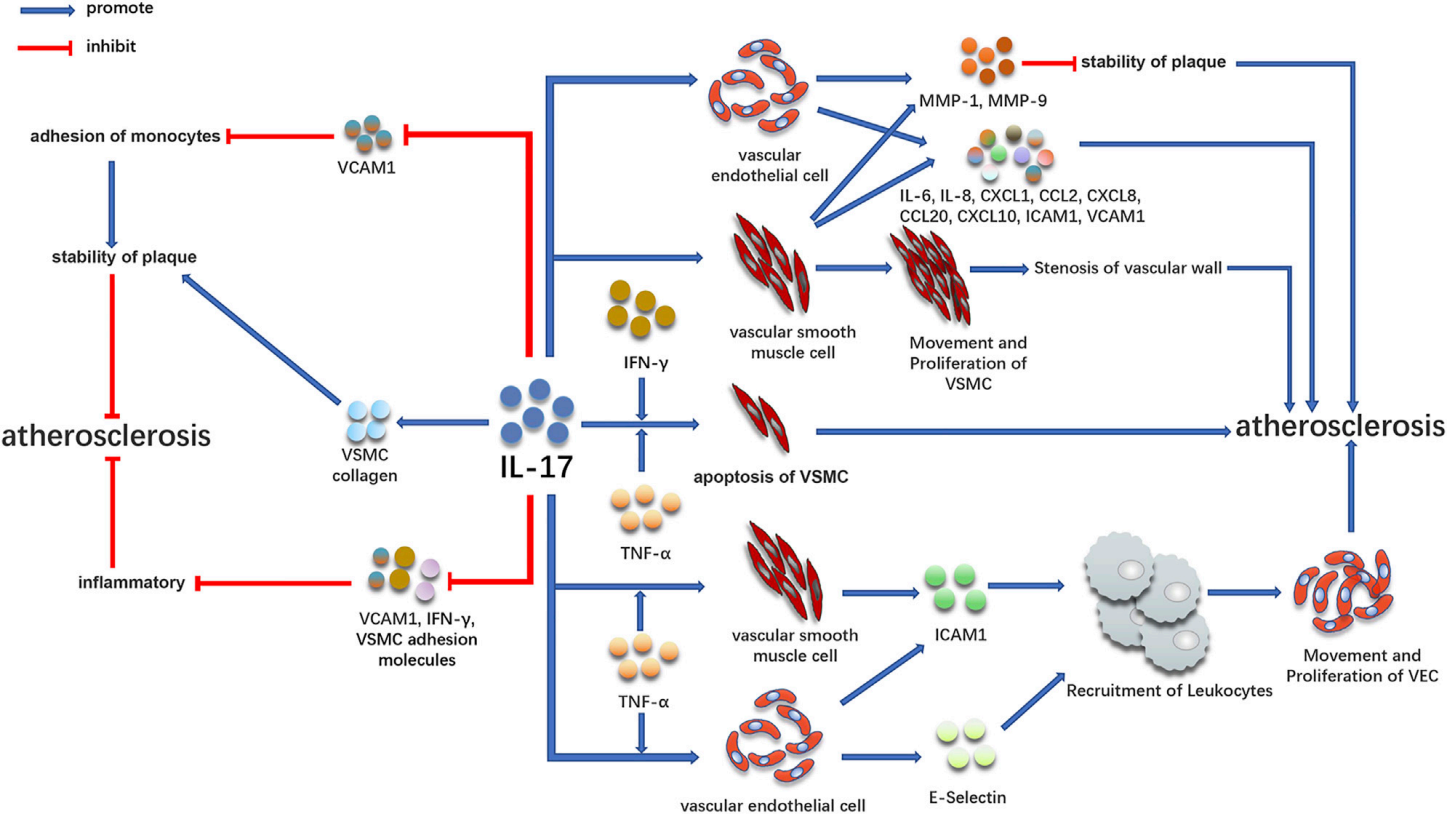
Promoting and inhibiting effect of IL-17 in atherosclerosis. IL-17 is involved in the development of atherosclerosis by acting on vascular endothelial cells and VSMC. At the same time, IL-17 can play an anti-atherosclerotic effect by increasing plaque stability and inhibiting inflammation, which may be related to the staging of the disease.

Early atherosclerotic CAD is associated with IL-17 (Figure 2). IL-17 is a cytokine that acts on blood vessels and cardiomyocytes and aggravates inflammation, coagulation, and thrombosis (Robert and Miossec, 2017). IL-17 can activate many downstream signaling pathways, including NF-KB, resulting in the expression of numerous pro-inflammatory cytokines (Iwakura et al., 2011). By binding to IL-17R on cells, IL-17 activates NF-KB under the action of conjugate protein ACT1 (Qian et al., 2007), causing the expression of different inflammatory factors, such as TNF-α, IL-6, IL-1β, monocyte chemoattractant protein-1, and intracellular adhesion molecule-1 (Feldmann et al., 2005; Liu et al., 2013; Soltanzadeh-Yamchi et al., 2018). These cytokines are involved in the formation and development of atherosclerotic plaque (Maracle et al., 2018; Song et al., 2018; Zhang et al., 2018). In the early stage, many animal models also observed direct evidence of the atherosclerotic effect of IL-17 (Erbel et al., 2009; van Es et al., 2009; Gao et al., 2010; Butcher et al., 2012; Zhang et al., 2012; Nordlohne and von Vietinghoff, 2019). Th17 cells play an essential role in the development of atherosclerotic plaque in mice, which may also affect atherosclerotic patients (Gao et al., 2010). By injecting IL-17 blocking antibody into apolipoprotein E deficient (apoE^−/−^) mice, it was discovered that functional blocking of IL-17 could decrease atherosclerotic lesions and improve as well as decrease plaque vulnerability, cell infiltration, and tissue activation, proposing that IL-17 plays a vital role in atherosclerosis formation (Erbel et al., 2009).

### IL-17 Regulates VSMC and Vascular Endothelial Cells, Leading to Atherosclerosis

The inflammatory reaction and proliferation of VSMC are the reasons for the progression of atherosclerosis (Chistiakov et al., 2015; Bennett et al., 2016). VSMC is mainly located in the middle layer of the vascular wall and plays a crucial role in atherosclerosis through proliferation, migration, apoptosis, and aging (Bennett et al., 2016). Under normal circumstances, VSMC in the arterial wall exhibits a contractile phenotype. When blood vessels are destroyed, VSMC exhibits proliferative and pro-inflammatory effects when it differentiates into a pro-inflammatory phenotype. Therefore, inflammation and excessive proliferation of VSMCs are the reasons for atherosclerosis progression (Chistiakov et al., 2015; Bennett et al., 2016). Persistent inflammatory stimulation plays a crucial role in atherosclerosis by promoting the proliferation and migration of VSMCs. IL-17 can cause the proliferation and migration of VSMCs dependent on NF-KB activator 1 (TRAF3IP2) and upregulate the expression of many mediators involved in angiogenesis and occlusive disease (Mummidi et al., 2019). IL-17 induces the production of pro-inflammatory cytokines (such as IL-6), chemokines (such as IL-8, CXCL-1, CCL-2, CXCL8, and CXCL10), and adhesion molecules (intercellular adhesion molecules 1ICAM1 and vascular cell adhesion molecules 1VCAM1) in vascular endothelial cells and VSMC (Fossiez et al., 1996; Eid et al., 2009; Erbel et al., 2009; Zhu et al., 2011; Erbel et al., 2014; Yuan et al., 2015), leading to atherosclerosis. Additionally, IL-17 can enhance the effect of TNF-α, further increase the expression of VCAM1 and ICAM1, and secrete IL-6, IL-8, and CCL20 by endothelial cells (Erbel et al., 2009; Hot et al., 2012). IL-17 may also aggravate atherosclerosis by increasing the expression of MMP1 and MMP9 and apoptosis of VSMCs and endothelial cells (Erbel et al., 2009; Zhu et al., 2011).

### IL-17 Combined With Other Cytokines Mediates the Occurrence of Atherosclerosis

IL-17 can also play a role with other inflammatory factors in inducing atherosclerosis. In the clinical specimens of coronary atherosclerosis, IL-17 and IFN-γ exist simultaneously, and T-cells can secrete IL-17 and IFN-γ in coronary plaques. IL-17 and IFN-γ jointly induce the release of IL-6, CXCL1, CXCL2, CXCL5, CCL8, and CXCL10 from VSMC (Eid et al., 2009). These chemokines play an essential role in the stability and activation of various cell types (Rolin and Maghazachi, 2014); different chemokines are also involved in the occurrence of atherosclerosis in different ways (Weber and Noels, 2011; Lu, 2017). Additionally, IFN-γ is highly expressed in atherosclerotic lesions and inhibits the proliferation of VSMC. While reducing collagen production, it makes the fibrous cap thinner by many expressions of MMPs, resulting in plaque rupture more easily (Harvey and Ramji, 2005; Tedgui and Mallat, 2006). The synergistic effect of IFN-γ and TNF-α can promote the formation of atherosclerosis (Mehta et al., 2017); IL-17 combined with TNF-α or IFN-γ leads to atherosclerosis by accelerating the apoptosis of VSMC (Clarke et al., 2008; Erbel et al., 2009). *In vitro* studies have indicated that IL-17 combined with TNF-α has a strong effect on promoting coagulation and thrombosis of vascular endothelial cells (Hot et al., 2012). Additionally, the combination of IL-17 and TNF-α can significantly increase E-selectin and intercellular adhesion molecules and promote the recruitment of leukocytes to form an inflammatory environment that causes the proliferation, migration, and invasion of endothelial cells (Robert and Miossec, 2017). These results suggest that IL-17 may promote the progression of atherosclerosis by inducing vascular inflammation, leukocyte recruitment, and plaque vulnerability.

### IL-17 has an Anti-atherosclerotic Effect (Figure 2)

However, it is worth noting that IL-17 can also promote the production of VSMC collagen, promote plaque stabilization, and inhibit the pathogenesis of atherosclerosis (Figure 2) (Erbel et al., 2009; Taleb et al., 2009; Smith et al., 2010; Gisterå et al., 2013; Hansson et al., 2015; Robert and Miossec, 2017). Some studies have indicated that IL-17 can inhibit the role of vascular cell adhesion molecules, fibroblasts, and VSMC adhesion molecules as well as decrease the production of IFN-γ, indicating that it has anti-inflammatory effects (Figure 2) (Madhur et al., 2010; Danzaki et al., 2012). Animal experiments have indicated that IL-17 exhibits resistance to low-density lipoprotein receptor^−/−^mouse atherosclerosis by improving the stability of plaques (Gisterå et al., 2013). The anti-atherosclerotic effect of IL-17 could be caused by inducing VSMC proliferation and downregulating the expression of VCAM1 in endothelial cells to inhibit the adhesion of monocytes to plaques to maintain the stability of plaques (Figure 2). Similarly, in a study of 981 patients with myocardial infarction, the high IL-17 expression in serum was significantly associated with low mortality and risk of recurrent myocardial infarction (Simon et al., 2013). VCAM1, as a biomarker of atherosclerosis, also exhibited a decreasing trend in these patients. The atherogenic and anti-atherosclerotic effects of IL-17 may be caused by the expression of IL-17 in various stages of the disease, in which the expression of IL-17 is highest in the early stage of atherosclerosis and reduced in the late stage of atherosclerosis.

## Summary and Prospect

RA is an autoimmune disease significantly associated with the increased risk of atherosclerosis (Mellado et al., 2015). The leading cause of death of patients with RA is cardiovascular disease. Chronic inflammation seems to be the main potential pathogenic factor connecting RA and cardiovascular disease. However, the mechanism of the link between RA and cardiovascular disease is not fully understood. In this review, by summarizing the mechanism of IL-17 in the pathogenesis of RA and atherosclerosis, it was found that autoantibodies activate the immune system and cells manufacture many cytokines and chemokines to activate dendritic cells owing to the reduced self-tolerance of the immune system in patients with RA. IL-23 expressed by activated dendritic cells mediates the differentiation of T-cells into TH17, which results in the expression of IL-17. IL-17 plays a vital role in the expression of adhesion molecules, pro-inflammatory cytokines and chemokines, cartilage and bone hyperplasia, bone destruction, and the proliferation, migration, and apoptosis of vascular endothelial cells, as well as VSMC. Simultaneously, the levels of IL-17 and IL-17-expressing cells in the serum of patients with RA and atherosclerosis exhibited an increasing trend. What is essential is that autoimmune diseases and atherosclerosis have common pathogenesis, and IL-17 plays a major role in the pathogenesis of autoimmune diseases and atherosclerosis, which promotes the secretion of different cytokines and aggravates the disease process to some extent.

There is increasing evidence that the morbidity and mortality of CVS are increasing in patients with RA. When formulating treatment plans for autoimmune diseases, such as RA, attention should be paid not only to relieving patients’ existing symptoms and reducing the injury of diseased joints but also to preventing cardiovascular diseases, especially the occurrence of RA, to decrease mortality to the greatest extent. IL-17 may be an essential index for the early diagnosis of RA complicated with atherosclerosis. In the early stage of RA, through the detection of IL-17, cardiovascular disease could be diagnosed in time and interventions could be initiated. IL-17 may become a potential target in treatment. Taking appropriate measures to decrease the expression of IL-17 or prevent it from binding to the receptor to reduce the symptoms of RA and decrease the possibility of atherosclerosis may be a feasible direction for treating RA complicated with cardiovascular disease. However, some studies have also suggested that IL-17 has an anti-atherosclerotic effect. So what is the reason that IL-17 has atherosclerotic and anti-atherosclerotic functions, and will this double-edged sword function change our view on the link between autoimmune and cardiovascular diseases? Will it provide us with new ideas for the prevention and treatment of these diseases? This will be our next research direction.
